# Description of genome sequences of arthropod-associated spirochetes of the genus *Entomospira*

**DOI:** 10.1128/mra.00740-24

**Published:** 2024-11-15

**Authors:** Silvie Sikutova, Lucía Graña-Miraglia, Marie Vancová, Tomáš Bílý, Andreas Sing, Santiago Castillo-Ramirez, Ivo Rudolf, Gabriele Margos, Volker Fingerle

**Affiliations:** 1 Institute of Vertebrate Biology, v.v.i., Czech Academy of Sciences, Brno, Czech Republic; 2 Cell and Systems Biology Department, University of Toronto, Toronto, Canada; 3 Biology Centre, Czech Academy of Sciences, Institute of Parasitology, České Budějovice, Czech Republic; 4 Faculty of Science, University of South Bohemia in Česke Budějovice, České Budějovice, Czech Republic; 5 National Reference Center for Borreliosis, Bavarian Health and Food Safety Authority, Oberschleissheim, Germany; 6 Programa de Genómica Evolutiva, Centro de Ciencias Genómicas, Universidad Nacional Autónoma de México, Cuernavaca, Morelos, Mexico; California State University, San Marcos, California, USA

**Keywords:** spirochaetes, *Entomospira *genus, mosquito, genome analysis

## Abstract

Spirochetal bacteria isolated from arthropods of the genera *Culex* and *Aedes* are termed BR149, BR151 (*Entomospira culicis*), BR193 (*Entomospira entomophila*), and BR208 (*Entomospira nematocerorum*). Genome sizes assembled from Illumina MiSeq and Oxford Nanopore reads varied between 1.67 and 1.78 Mb containing three to six plasmids. GC content ranged from 38.5% to 45.76%.

## ANNOUNCEMENT

Unique spirochetal strains were isolated from mosquitoes and flies between 1999 and 2002 in the Czech Republic (South Moravia) using standard entomological (an aspirator for adults) and hydrobiological (dipper for larvae) equipment and described as a new genus *Entomospira* (1, 2). Among spirochaetal bacteria, the genus *Borrelia* is known for having numerous plasmids, and even with next-generation sequencing techniques, plasmid assembly issues have been noticed (3, 4). Although many genomes of spirochaetal bacteria are available in GenBank (www.ncbi.nlm.nih.gov/genome/?term=Spirochaetales), information on plasmids is scarce. Here, we used recently established methods (3) and additional software tools to re-evaluate the genomes of species of the genus *Entomospira* with particular emphasis on plasmids.

Four isolates (BR149, BR151, BR193, and BR208) were isolated and grown in BSK-H at 33°C (initial cultures used BSK-H medium containing the antibiotics phosphomycin 100 µg/mL and rifampicin 50 µg/mL, Sigma) (1). DNA was extracted using a Qiagen DNA Minikit (Qiagen, Germany) and sequenced on an Illumina MiSeq (kits: library preparation TG-131-1096, indexing FC-131-100, sequencing MS-102-2002, paired-end read length 150 bp; adapter trimming via Illumina pipeline; read quality assessment: fastqc v0.12.1; Illumina, Germany) and an Oxford Nanopore MinION [SQK-PBK004, library preparation according to the manufacturer’s recommendations with DNA shearing via gtubes (Covaris, UK), run time 16 h, flowcell 9.6, Oxford, UK; average read length: 1,172 bp (BR149), 1,897 bp (BR151), 2,584 bp (BR193), and 2,717 bp (BR208)] using Albacore 2.0 for demultiplexing and base calling. Library quality check: 2200 Tapestation (Agilent, Germany). Hybrid genome assemblies using SPAdes 3.9.0 (5) revealed a spirochetal genome consisting of a chromosome and three to six plasmids. The quality of genomes was evaluated using CheckM 1.1.0 (6). The presence, topology, and completeness of plasmids were evaluated using Platon 1.7.0 (7) and the online version of BLASTn 2.14.0 and nucleotide collection (nr/nt) database (8). Overlapping ends of contigs visible in dot plots or specified in alignments indicated circularity and completeness (3). For assembly data and GenBank accession numbers, see Table 1.

**TABLE 1:** Assembly and genomic features of four *Entomospira* isolates

Isolate features	BR149	BR151	BR193	BR208
Host	*Culex pipiens*	*Culex pipiens*	*Aedes cinereus*	*Culex pipiens*
Year of isolation	1999	1999	2000	2001
Isolation site	South Moravia, Czech Republic	South Moravia, Czech Republic	South Moravia, Czech Republic	South Moravia, Czech Republic
Environmental medium	Mosquito midgut	Larval mosquito midgut	Adult female mosquito midgut	Larval mosquito midgut
Number of contigs	8	13	9	7
N50 value	1,639,683	427,344	1,486,042	1,418,720
Coverage	146	88	104	91
Average GC content (%)	45.8	45.8	40.4	38.5
Chromosome size	1.6 Mbp	1.6 Mbp	1.49 Mbp	1.68 Mbp
Chromosome GenBank accession	CP118185	CP118181	CP118174	CP118168
Number of plasmids	3	3	6	5
Sizes of plasmids (bp)	98,591^*a*^ 28,402^*a*^ 3,028	98,591^*a*^ 28,402^*a*^ 3,005	118,941^*a*^ 107,998^*a*^ 41,864 22,271^*a*^ 22,271^*a*^ 6. 3,708^*a*^	110,286^*a*^ 53,611^*a*^ 38,620 37,695^*a*^ 21,443^*a*^
Plasmid GenBank accession	CP118186 CP118187 CP118188	CP118182 CP118183 CP118184	CP118175 CP118176 CP118177 CP118178 CP118179 CP118180	CP118169 CP118170 CP118171 CP118172 CP118173
Genes (total)	1,674	1,675	1,736	1,586
Genes (protein-coding)	1,622	1,623	1,685	1,536
Encoding hypothetical proteins	506	508	546	447
Genes (RNA)	47	47	45	46
rRNAs (5S, 16S, 23S)	2, 2, 2	2, 2, 2	1, 2, 1	2, 2, 1
tRNAs	41	41	41	41
SRA Illumina	SRX24846788	SRX24846787	SRX24846789	SRX24846790
SRA ONT	SRX24846792	SRX24846791	SRX24846793	SRX24846794

^*a*^ Plasmids were confirmed using the software Platon 1.7.0 (7).

**Fig 1:**
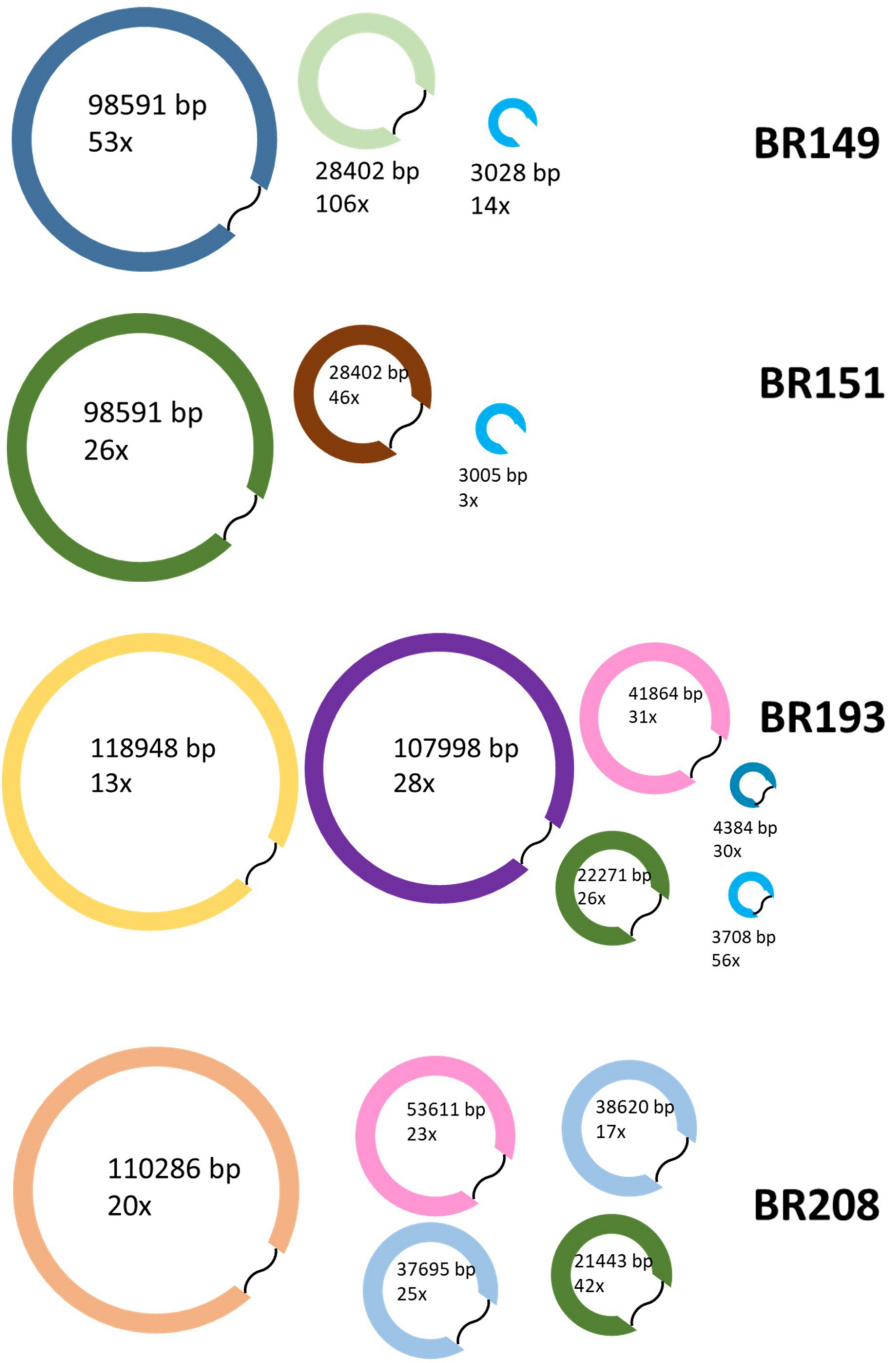
Visualization of plasmids found in the genus *Entomospira*. To determine the structure and size of plasmids, sequence data were analyzed using online BLASTn 2.14.0 (8), Platon 1.7.0 (7), plasmidSPAdes 3.9.0 (10), and Bandage 0.8.1 (9). Size (in bp) and coverage (x) are given inside or underneath the drawings. Most plasmids appeared to be circular except a contig of 3 kbp found in BR149 and BR151 for which insufficient information was available. Sizes are not according to scale. Image modified from reference (2).

The genome sizes of BR149 and BR151 were 1.76 Mbp, of BR193 1.78 Mbp, and of BR208 1.68 Mpb. Mol% G+C values ranged between 38.5 and 45.8 (Table 1). Chromosome sizes, plasmid number, sizes, and coverage are given in Table 1; Fig. 1. One contig of approximately 3 kbp (BR149, BR151) did not have overlapping ends suggesting that this plasmid was either incompletely assembled or may not be a plasmid at all. Low coverage underlines this (Fig. 1). Genome annotation of BR149 and BR151 in the NCBI Prokaryotic Genome Annotation Pipeline (PGAP v.6.4) showed that the genomes contained at least 1,622 coding sequences (CDS) of which 31% encode hypothetical proteins. Although the contig of 41,864 bp in BR193 was shown as a plasmid in an assembly graph using Bandage 0.8.1 (9), had a reasonable coverage, and showed overlapping ends, it was not confirmed by plasmid analysis in Platon (7). Annotation of the BR193 genome indicated 1,685 CDS; 546 (32%) encode hypothetical proteins. In BR208, the plasmid of 38 kbp was not confirmed by Platon (7). The BR208 genome has 1,536 CDS of which 29% (447) encode hypothetical proteins.

## Data Availability

Cultures are available in the Deutsche Sammlung von Mikroorganismen und Zellkulturen (German Collection of Microorganisms and Cell Cultures), Germany, https://www.dsmz.de/, DSMZ (BR151 = DSM 114560^T^, BR193 = DSM 114561^T^, BR208 = DSM 114562^T^), and the Netherlands Culture Collection of Bacteria Westerdijk Institute, The Netherlands, https://wi.knaw.nl/Collection, NCCB (BR151 = NCCB 100891^T^, BR193 = NCCB 100892^T^, BR208 = NCCB 100893^T^). 16S rRNA sequences are available in NCBI GenBank under the following accession numbers: for BR151, ON426176; for BR193, ON426177; and for BR208, ON426178. Genome sequences are available in NCBI GenBank under the following accession numbers: for Entomospira culicis BR149, Illumina SRX24846788, ONT SRX24846792, chromosome CP118185, and plasmids CP118186-CP118188; for Entomospira culicis BR151, Illumina SRX24846787, ONT SRX24846791, chromosome CP118181, and plasmids CP118182-CP118184; for Entomospira entomophila BR193, Illumina SRX24846789, ONT SRX24846793, chromosome CP118174, and plasmids CP118175-CP118180; and for Entomospira nematocerorum BR208, Illumina SRX24846790, ONT SRX24846794, chromosome CP118168, and plasmids CP118169-CP118173.
